# Evaluation of the Ability of LL-37 to Neutralise LPS *In Vitro* and *Ex Vivo*

**DOI:** 10.1371/journal.pone.0026525

**Published:** 2011-10-18

**Authors:** Aaron Scott, Sinéad Weldon, Paul J. Buchanan, Bettina Schock, Robert K. Ernst, Danny F. McAuley, Michael M. Tunney, Chris R. Irwin, J. Stuart Elborn, Clifford C. Taggart

**Affiliations:** 1 Centre for Infection and Immunity, School of Medicine, Dentistry and Biomedical Sciences, Queen's University Belfast, Belfast, Northern Ireland; 2 Department of Microbial Pathogenesis, School of Dentistry, University of Maryland, Baltimore, Maryland, United States of America; 3 School of Pharmacy, Queen's University Belfast, Belfast, Northern Ireland; 4 Centre for Dental Education, School of Medicine, Dentistry and Biomedical Sciences, Queen's University Belfast, Belfast, Northern Ireland; University of California Merced, United States of America

## Abstract

**Background:**

Human cathelicidin LL-37 is a cationic antimicrobial peptide (AMP) which possesses a variety of activities including the ability to neutralise endotoxin. In this study, we investigated the role of LPS neutralisation in mediating LL-37's ability to inhibit *Pseudomonas aeruginosa* LPS signalling in human monocytic cells.

**Methodology/Principal Findings:**

Pre-treatment of monocytes with LL-37 significantly inhibited LPS-induced IL-8 production and the signalling pathway of associated transcription factors such as NF-κB. However, upon removal of LL-37 from the media prior to LPS stimulation, these inhibitory effects were abolished. These findings suggest that the ability of LL-37 to inhibit LPS signalling is largely dependent on extracellular LPS neutralisation. In addition, LL-37 potently inhibited cytokine production induced by LPS extracted from *P. aeruginosa* isolated from the lungs of cystic fibrosis (CF) patients. In the CF lung, polyanionic molecules such as glycosaminoglycans (GAGs) and DNA bind LL-37 and impact negatively on its antibacterial activity. In order to determine whether such interactions interfere with the LPS neutralising ability of LL-37, the status of LL-37 and its ability to bind LPS in CF sputum were investigated. Overall our findings suggest that in the CF lung, the ability of LL-37 to bind LPS and inhibit LPS-induced IL-8 production is attenuated as a result of binding to DNA and GAGs. However, LL-37 levels and its concomitant LPS-binding activity can be increased with a combination of DNase and GAG lyase (heparinase II) treatment.

**Conclusions/Significance:**

Overall, these findings suggest that a deficiency in available LL-37 in the CF lung may contribute to greater LPS-induced inflammation during CF lung disease.

## Introduction

Cystic fibrosis (CF) is an autosomal recessive disease caused by mutations in the cystic fibrosis transmembrane conductance regulator (CFTR). Mutated CFTR results in defective electrolyte transport in the airways which leads to a continual cycle of airway surface liquid dehydration, airway obstruction, chronic bacterial infection and airway inflammation [1], [2]. Research to date suggests that mutations in the CFTR gene contribute to the dysregulation of a variety of components of the innate immune system [3], [4]. Antimicrobial peptides (AMPs) play an essential role in the innate immune system and contribute to host defence through direct antimicrobial activity, as well as by modulating innate and adaptive immunity and wound repair [5]. In CF, the antibacterial capacity of airway fluid is paradoxically low despite the presence of a high AMP load [6]–[8]. Levels of one AMP in particular, the human cathelicidin LL-37, are significantly elevated in CF lung secretions and levels correlated with disease severity in CF patients [9], [10]. Research to date supports the hypothesis that despite being present in large quantities in the CF airways, the antimicrobial activity of LL-37 is markedly restricted although the nature of this defect *in vivo* remains unclear.

LL-37 has a broad spectrum of antimicrobial activity acting against both Gram-positive and Gram-negative bacteria including *Staphylococus aureus* and *Pseudomonas aeruginosa* [11]–[16]. In addition, a low concentration of LL-37 (0.5 µg/ml) potently inhibited the formation of *P. aeruginosa* biofilm *in vitro* [17]. As previously mentioned, the antimicrobial activity of LL-37 is markedly restricted in the CF lung. The results to date have suggested both a salt-dependent [6] and -independent [8], [15] restriction of antimicrobial activity. Elevated levels of LL-37 in CF are thought to be a consequence of increased local production and correlate with neutrophilia – a characteristic of the CF airways [9], [10]. Nevertheless, the anionic poly-electrolytes DNA and filamentous (F)-actin derived mainly from neutrophils contribute significantly to viscosity in the CF lung and strongly inhibit the antibacterial activity of LL-37 against *P. aeruginosa* [18], [19]. In addition, previous work has found that the antibacterial activity of LL-37 was inhibited by a range of molecules including bacterial polysaccharides [20], [21], mucins [22], [23], proteases [24], in addition to glycosaminoglycans (GAGs) present in wound fluids [25] and CF lung secretions [26]. Treatment of CF lung secretions *in vitro* with gelsolin, polyanions such as poly-aspartate, DNase and GAG lyases, and *in vivo* with nebulised hypertonic saline [19], [26] are reported to increase the levels of LL-37 as well as the bactericidal activity of samples.

LL-37 possesses activities extending beyond its basic bactericidal activity and a large body of work has focused on the immunomodulatory activity of LL-37, which may be as, or more, important than its direct antimicrobial action under physiological conditions [23], [27]. Lipopolysaccharide (LPS) neutralising activities of LL-37 have been well characterised *in vitro* and *in vivo* [28]–[32]. Although previous research has focussed on the bactericidal activity of LL-37 in CF lung secretions and its inhibition by molecules such as DNA and GAGs, the effects of such molecules on the ability of LL-37 to neutralise/inhibit LPS signalling are unknown. *P. aeruginosa* LPS is a prominent factor in mediating both bacterial virulence and host responses in susceptible individuals such as CF patients [33]. Therefore, the aim of this study was to investigate the importance of LPS neutralisation in the anti-endotoxin activity of LL-37 and to determine the efficacy of this important biological effect in CF lung secretions.

## Materials and Methods

### Ethics Statement

Ethical approval was received from the Office for Research Ethics Northern Ireland (ethical approval study number 06/NIR01/11) with all patients providing written informed consent prior to participation.

### Materials

Recombinant human LL-37 and rabbit anti-LL-37 were purchased from Innovagen (Lund, Sweden). Complete protease inhibitor cocktail tablets were from Roche Diagnostics. LPS from *P. aeruginosa* (serotype 10) and heparinase II from *Flavobacterium heparinum* were purchased from Sigma-Aldrich, Dorset, UK. Mouse anti-phospho IκBα (Ser32/36), rabbit anti-IκBα, anti-phospho IKKα/β (Ser180/181) were purchased from Cell Signaling Technology, Danvers, Massachusetts, USA. Rabbit anti-IκBβ and anti-GAPDH were purchased from Santa Cruz Biotechnology, Germany. HRP-conjugated secondary antibodies were obtained from Thermo Fisher Scientific, Northumberland, UK. All other reagents were of analytical grade and were purchased from Sigma-Aldrich unless otherwise stated.

### *P. aeruginosa* LPS Preparation and Mass Spectrometry Procedures

LPS was extracted from *P. aeruginosa* strains isolated from the lungs of two CF patients with severe lung disease (SE4 and SE22) and lipid A structures analysed by negative ion matrix-assisted laser desorption ionization time-of-flight (MALDI-TOF) mass spectrometry (MS). LPS was purified by Mg^2+^–ethanol precipitation as described by Darveau and Hancock [34] after growth in lysogenic broth supplemented with 1 mM MgCl_2_ at 37°C. Individual LPS samples were additionally extracted to remove contaminating phospholipids [35] and TLR2 contaminating proteins [36]. Lipid A was isolated after hydrolysis in 1% SDS at pH 4.5 as described [37]. Briefly, 500 µl of 1% SDS in 10 mM Na-acetate, pH 4.5 was added to a lyophilized sample. Samples were incubated at 100°C for 1 h, frozen, and lyophilised. The dried pellets were resuspended in 100 µl of water and 1 ml of acidified ethanol (100 µl 4 N HCl in 20 ml 95% EtOH). Samples were centrifuged at 5,000 rpm for 5 minutes. The lipid A pellet was further washed (3×) in 1 ml of 95% EtOH. The entire series of washes was repeated twice. Samples were resuspended in 500 µl of water, frozen on dry ice and lyophilised. MALDI-TOF MS experiments were performed as described for the analysis of LPS or lipid A preparations with the following modifications [38]. Lyophilized lipid A was dissolved with 10 µl 5-chloro-2-mercaptobenzothiazole (CMBT) MALDI matrix in chloroform/methanol, 1∶1 (v/v), and then applied (1 µl) onto the sample plate. All MALDI-TOF experiments were performed using a Bruker Autoflex III MALDI-TOF mass spectrometer (Bruker Daltonics Inc., Billerica, MA). Each spectrum was an average of 200 shots. ES Tuning Mix (Agilent, Palo Alto, CA) was used to calibrate the MALDI-TOF MS. Commercially available *P. aeruginosa* LPS (serotype 10) has a penta-acylated structure (data not shown). In contrast, SE4 and SE22 isolates from CF patients synthesise hexa- and hepta-acylated LPS, respectively [39].

### Cell culture and treatment

THP-1 human acute monocytic leukemia cell line was obtained from the European Collection of Cell Cultures and cultured routinely in RPMI supplemented with 10% FBS, l-glutamine, penicillin and streptomycin (Invitrogen, Paisley, UK). Unless otherwise stated, cells were pre-treated with LL-37 (10 µg/ml) for 1 h before stimulation with LPS for 24 h for ELISA. For Western blotting experiments, cells were pre-treated with LL-37 for 1 h and subsequently stimulated with LPS for 0–120 min as indicated in the figure legends. In some experiments, cells were washed with PBS after the 1 h pre-treatment with LL-37 and then stimulated with LPS as indicated.

### Western blotting

THP-1 cells were treated as described above and cytoplasmic extracts were prepared as described previously [40]. Protein concentrations of extracts were determined and equal concentrations of samples were separated on 10% SDS-polyacrylamide gels and transferred onto 0.2 µm nitrocellulose membrane. The membrane was blocked for 1 h at room temperature with 5% Marvel or 3% BSA in PBS containing 0.1% Tween 20 (PBST) according to the antibody manufacturer's instructions. Cytoplasmic extracts were immunoblotted for IκBα, IκBβ and phosphorylated forms of IκBα (Ser32/36) and IKKα/β (Ser180/181). Equal loading of samples was confirmed by immunoblotting for GAPDH. Proteins were detected using the appropriate HRP-conjugated secondary antibody and visualised by chemiluminescence (GE Healthcare UK, Buckinghamshire, UK) and analysed using the Syngene GeneSnap and GeneTools software on the ChemiDoc system.

### IL-8 ELISA

IL-8 levels in cell-free supernatants were quantified using a commercially available ELISA kit (R&D Systems) according to manufacturer's instructions.

### Sputum processing

Expectorated sputum samples were obtained from CF patients (n = 12) under the Office for Research Ethics Northern Ireland ethical approval study number 06/NIR01/11. Each sample was suspended in 8× volumes of saline with 1 M DTT and placed on a roller for 1 h to allow full disruption of the dehydrated mucus. The samples were then centrifuged at 13,000 rpm for 20 min and the resultant supernatant stored at −80°C until required for analysis.

### Sulphated glycosaminoglycan (sGAG) assay

GAG levels were measured using the Blyscan Blue sGAG assay as directed by the manufacturer (Biocolour Ltd., Co Antrim, Northern Ireland). Briefly, sputum and standards were made up as directed and incubated with 1 ml of Dye Reagent (1,9-dimethy-methylene blue) for 30 min at room temperature with constant agitation. Samples were spun at 13000× g for 10 min to pellet resulting precipitate, which was resuspended in 1 ml of Dissociation Reagent for 10 min. Samples and standards (200 µl) were added to a 96 well plate and the absorbance determined at 656 nm and results were expressed as µg/ml.

### DNase/ heparinase II treatment of CF sputum

CF sputum was incubated with Pulmozyme® (dornase alfa; recombinant human DNase I, Genentech, USA) and the lyase heparinase II to cleave DNA fragments and sulphated GAGs, respectively. CF sputum was incubated with DNase (30 µg/ml) and/or heparinase II (10 mU) for 1 h at 37°C.

### LL-37 ELISA

Total LL-37 levels were measured in treated and untreated CF sputum by indirect ELISA as previously described with some modifications [41]. LL-37 standards/samples were added to Greiner® high binding 96 well plates and dried overnight at 37°C. Plates were washed 3 times with PBST and blocked in PBST containing 1% BSA for 1 h at room temperature. Rabbit anti-LL-37 antibody was added to the plate for 2 h (100 µl/well, 1∶10,000). Plates were washed 3 times with PBST and 100 µl of HRP-conjugated goat anti-rabbit antibody added per well for 1 h at room temperature. After washing, peroxidase activity was measured by the addition of ABTS substrate (Invitrogen) and reading the absorbance at 405 nm on a microtitre plate reader (Genios using Magellan software).

### LPS-binding assay

The ability of LL-37 present in treated and untreated CF sputum to bind LPS was analysed by ELISA as previously described with some modifications [42]. Briefly, Greiner® high binding 96 well plates were coated with 100 ng/well of LPS diluted in serum free media (SFM) and incubated at 37°C for at least 3 h. The wells were washed 3 times with distilled water before air-drying overnight at room temperature. Plates were blocked with 1% BSA in PBST for at least 1 h at room temperature. Standards and sputum samples were made up to 100 µl in SFM and incubated on the plate at 37°C for 2 h. All samples were loaded in duplicate and diluted in a 1∶2 dilution series. After washing 3 times with PBST, rabbit anti-LL-37 antibody was added to the plate for 2 h at room temperature (100 µl/well, 1∶10,000). Plates were again washed and 100 µl of HRP-conjugated goat anti-rabbit antibody added per well for 2 h at room temperature. Peroxidase activity was measured and quantified as described above.

### LPS Neutralisation Assay

The ability of LL-37 present in treated and untreated CF sputum to inhibit LPS-induced IL-8 production by THP-1 monocytic cells was analysed by a modification of the above LPS-binding ELISA protocol. LPS was immobilised in Greiner® high binding 96 well plates as described above. The wells were washed 3 times with SFM before samples were added. CF sputum samples (n = 12) were treated with Pulmozyme® DNase and/or heparinase II as described previously in the absence or presence of rabbit anti-LL-37 antibody (1 µg/ml) or heat-inactivated (HI) antibody as a negative control. Sputum samples were made up to 100 µl in SFM and incubated on the plate at RT for 1 h. After washing 3 times with SFM, THP-1 cells (1×10^6^/ml) were added to each well and incubated for 6 h at 37°C. Cells were then collected and IL-8 levels in cell free supernatants were determined by ELISA.

### Statistical Analyses

All statistical analyses were performed using GraphPad PRISM 5.0 software package (San Diego, CA). Results are expressed as the mean ± SEM and unless specified otherwise all results are representative of at least three independent experiments performed in duplicate. Differences between multiple treatments were compared by one-way analysis of variance (ANOVA) followed by Bonferroni's Multiple Comparison post test. A *p* value of <0.05 was considered to represent a statistically significant difference (* *p*<0.05; ** *p*<0.01; *** *p*<0.001).

## Results

### LL-37 exerts potent anti-endotoxin effects in THP-1 monocytes

**Figure 1:**
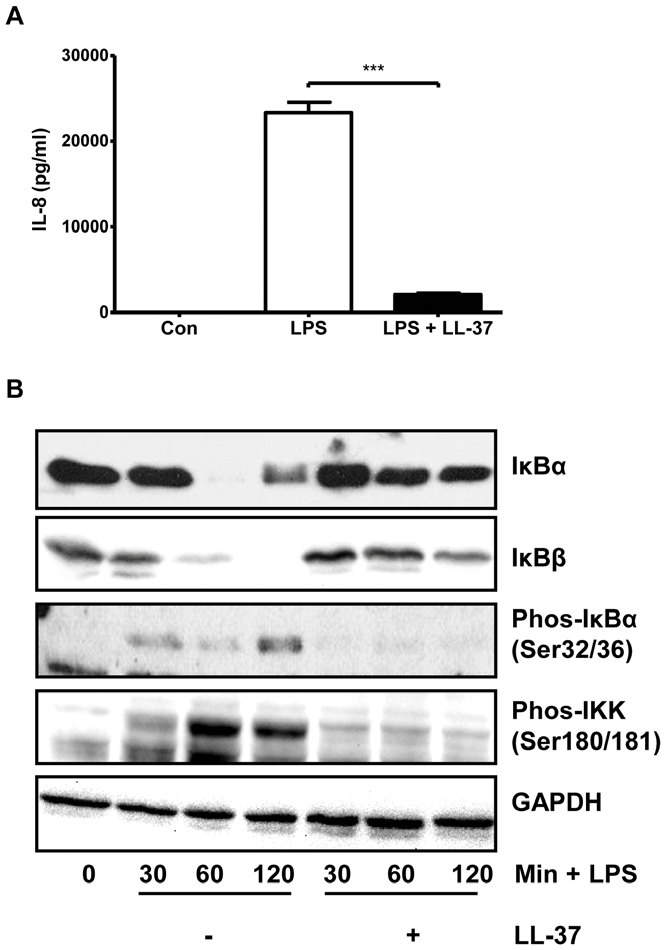
LL-37 inhibits LPS-induced IL-8 production and IκB degradation in THP-1 monocytic cells. (**A**) THP-1 monocytes were pre-treated for 1 h with 10 µg/ml LL-37 and incubated in the absence or presence of 1 µg/ml *P. aeruginosa* LPS. After 24 h stimulation with LPS, IL-8 levels were quantified in cell-free culture supernatants by ELISA. *** *p*<0.001; Con, control. (**B**) Cytoplasmic extracts were prepared following 30, 60 and 120 min and levels of IκBα, IκBβ, phosphorylation of IκBα (Ser32/36) and IKKα/β (Ser180/181) were determined by Western blotting. GAPDH was used to control for protein loading.

The ability of LL-37 to inhibit LPS-induced pro-inflammatory cytokine production is well documented. As shown in Figure 1A, pre-treatment with LL-37 significantly inhibited commercially available *P. aeruginosa* LPS-induced IL-8 production from THP-1 human monocytic cells by approximately 90%. Investigation of upstream regulators of IL-8 production revealed that pre-treating monocytes with LL-37 down-regulated the LPS-induced NF-κB pathway, as illustrated in Figure 1B. Stimulation of THP-1 cells with LPS resulted in the almost complete degradation of the inhibitory proteins IκBα and IκBβ by 60 and 120 min, respectively, compared to control cells. Pre-treatment of cells with LL-37 inhibited this degradation at corresponding time points. Degradation of IκB proteins in response to a pro-inflammatory stimulus such as LPS occurs after their phosphorylation, which is regulated by kinases such as IKKα/β. Phosphorylation of IκBα (Ser32/36) was observed following LPS stimulation and this phosphorylation was inhibited by LL-37. In addition, phosphorylation of IKKα/β (Ser180/181) was similarly induced by LPS stimulation compared to control cells, and again, this phosphorylation event was inhibited by LL-37 pre-treatment.

### Removal of exogenous LL-37 after pre-treatment diminishes its anti-inflammatory activities

**Figure 2:**
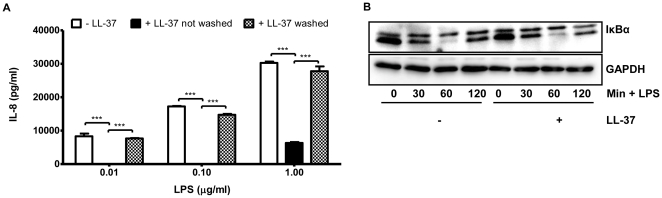
Removal of exogenous LL-37 prior to LPS stimulation diminishes its anti-endotoxin activity. (**A**) THP-1 monocytes were pre-treated for 1 h with LL-37 (10 µg/ml) and then washed with sterile PBS to remove extracellular, non-incorporated, exogenous LL-37. The cells were resuspended in fresh media and stimulated with 0.01, 0.1 and 1 µg/ml LPS for 24 h. IL-8 levels in cell-free culture supernatants were quantified by ELISA. (**B**) THP-1 monocytes were pre-treated for 1 h with 10 µg/ml LL-37 and washed as described previously. Following resuspension, cells were stimulated with 1 µg/ml LPS for 30, 60 and 120 min. Cytoplasmic extracts were prepared and IκBα levels were determined by Western blotting. GAPDH was used to control for protein loading.

In order to investigate whether the ability of LL-37 to inhibit LPS-induced signalling was dependent on its presence in the cell culture media at the time of LPS stimulation, experiments were performed in which THP-1 monocytes were pre-treated with LL-37 (10 µg/ml) for 1 h and then washed 3 times with sterile PBS to remove exogenous peptide. The cells were then stimulated with 0.01, 0.1 and 1 µg/ml LPS and IL-8 levels were measured in cell free supernatants after 24 h (Figure 2A). In contrast to the pronounced inhibition of LPS-induced IL-8 production we observed in Figure 1A, when the cells were washed to remove exogenous LL-37 prior to LPS stimulation no significant inhibition was observed when compared to LPS alone, regardless of concentration of LPS used (Figure 2A) of whether cells were pre-treated for 1 h or 2 h prior to washing (data not shown). In agreement with previous work, our results demonstrate that the sustained presence of LL-37 at the time of stimulation is necessary for maximal anti-endotoxin effect regardless of LPS concentration [43]. Furthermore, washing the cells prior to stimulation diminished the ability of LL-37 to inhibit LPS-induced IκBα degradation (Figure 2B) that was previously observed in Figure 1B.

### LL-37 potently inhibits IL-8 production induced by clinical *P. aeruginosa* LPS isolates from CF patients

Our findings to date highlight the importance of direct interaction between LPS and LL-37 in mediating its maximal anti-endotoxin activities in response to commercially available *P. aeruginosa* LPS formulations. Although, the contribution of LPS to pathogenesis varies depending on a number of factors, unique lipid A modifications that occur in clinical *P. aeruginosa* CF isolates are associated with CF lung infection [39], [44], [45]. TLR4-mediated responses are highly dependent on the level of acylation of the lipid A structure of LPS. *P. aeruginosa* synthesizes more highly acylated LPS structures during adaptation to the CF airway environment [34], [45]–[47]. Although such modifications can confer resistance to cationic AMPs such as C18G and polymyxin [44], their effect on the ability of LL-37 to neutralise the modified LPS is unknown. Therefore, to evaluate the effect of LL-37 on the IL-8 inducing ability of such LPS preparations, THP-1 monocytes were pre-treated with LL-37 (5 µg/ml) for 1 h prior to stimulation with LPS extracted from *P. aeruginosa* strains isolated from the lungs of two CF patients with severe lung disease (SE4 and SE22) for 24 h. Commercially available *P. aeruginosa* LPS (serotype 10) has a penta-acylated structure (data not shown), whereas SE4 and SE22 LPS isolates from CF patients synthesise hexa- and hepta-acylated LPS, respectively [39].

**Figure 3:**
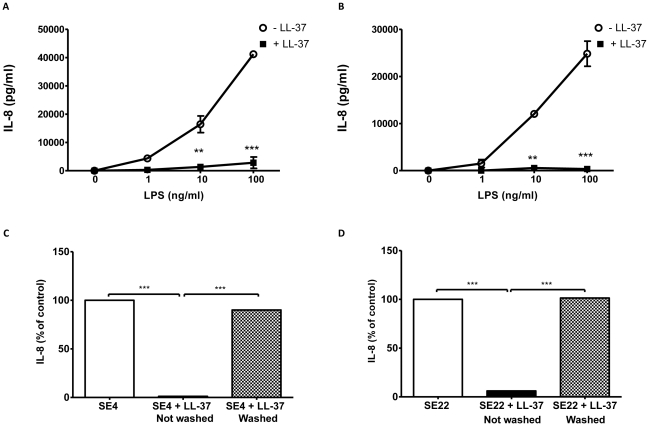
LL-37 neutralises LPS from clinical strains of *P. aeruginosa* isolated from CF patients with severe airway disease. THP-1 monocytes were pre-treated with 5 µg/ml LL-37 for 1 h before cells were stimulated for 24 h with (**A**) SE4 and (**B**) SE22 LPS isolates from CF patients with severe airway disease (1, 10 and 100 ng/ml). IL-8 levels in cell-free culture supernatants were quantified by ELISA. ** *p*<0.01; *** *p*<0.001 compared to corresponding LPS alone (- LL-37). THP-1 monocytes were pre-treated for 1 h with LL-37 (5 µg/ml) and then washed with sterile PBS to remove extracellular, non-incorporated, exogenous LL-37. The cells were resuspended in fresh media and stimulated with 50 ng/ml (**C**) SE4 and (**D**) SE22 LPS for 24 h. IL-8 levels in cell-free culture supernatants were quantified by ELISA. Results are expressed as percentage of control response (100% = LPS alone). *** *p*<0.001.

As shown in Figure 3A and 3B, both LPS preparations significantly induced THP-1 monocyte IL-8 production when compared to untreated control cells in a dose-dependent manner. LL-37 pre-treatment significantly inhibited induction of IL-8 by the LPS isolates at each concentration of LPS (>90%). These findings demonstrate that acylation of the lipid A component of *P. aeruginosa* LPS does not affect the ability of LL-37 to neutralise and inhibit LPS-induced IL-8 production from human monocytic cells. Similar to what was observed for commercial *P. aeruginosa* LPS in Figure 2A, there was no significant difference between LPS and LPS+LL-37 when the cells were washed following LL-37 pre-treatment (Figure 3C and 3D). In contrast, with continued presence of LL-37, IL-8 production was inhibited >90%. Overall, these findings again support previous observations that the sustained presence of LL-37 at the time of stimulation is necessary for maximal anti-endotoxin effect regardless of LPS source.

### GAGs and DNA present in the CF lung abrogate the ability of LL-37 to neutralise LPS

**Figure 4:**
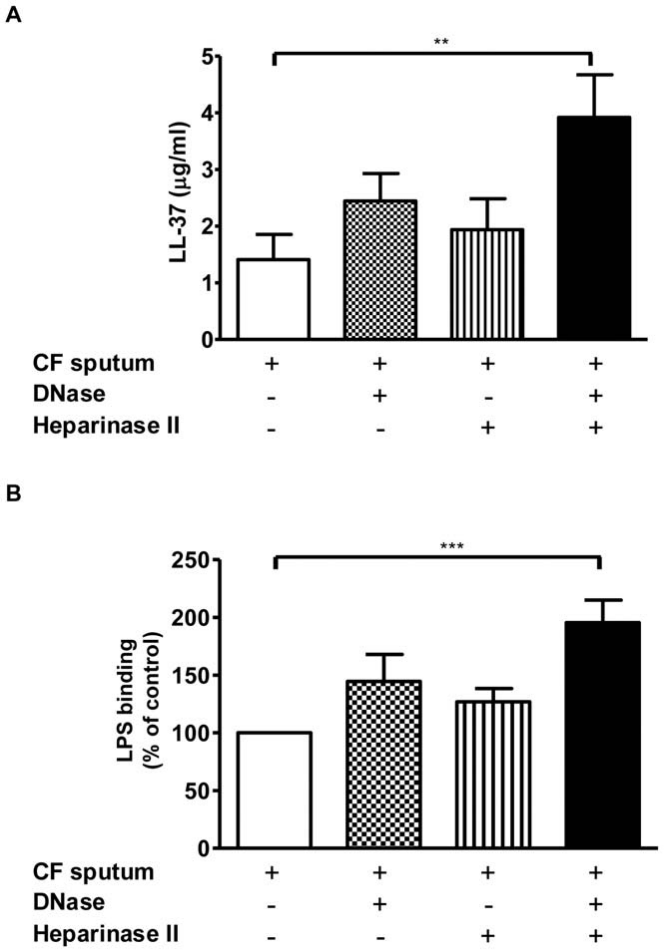
LL-37 levels and its ability to bind LPS in CF sputum are increased following treatment with DNase and heparinase II. (**A**) CF sputum samples (n = 12) were treated with Pulmozyme® DNase and/or heparinase II for 1 h at 37°C. The LL-37 content of these samples and untreated sputum samples were quantified by ELISA. (**B**) The ability of available LL-37 present in untreated and treated CF sputum samples to bind LPS was determined by ELISA. Results are expressed as percentage of untreated control (100% = untreated CF sputum). ** *p*<0.01; *** *p*<0.001.

LL-37 has previously been demonstrated to bind to GAGs and DNA and such interactions negatively impact on the antimicrobial activity of LL-37 [19], [26]. In agreement with previous data [48], [49], GAG levels were found to be significantly elevated in the CF lung (328±31 µg/ml) compared to healthy controls (not detectable). Given the importance of a physical interaction between LL-37 and LPS for the peptide to inhibit LPS signalling, we investigated the status and LPS-binding ability of LL-37 in CF sputa treated with DNase and/or the lyase heparinase II. Heparinase II was used to degrade polysaccharide GAGs as it possesses the broadest known substrate specificity of the heparinases [50]. Treatment with DNase or heparinase II alone induced a slight, increase in total LL-37 levels compared to untreated CF sputum (Figure 4A). However, a combination treatment of both DNase and heparinase II significantly increased the amount of LL-37 detectable in CF sputum when compared to untreated CF sputum samples. With this increase in liberated LL-37, a concomitant significant increase in LPS-binding was also observed (Figure 4B) in DNase- and heparinase II-treated CF sputum.

**Figure 5:**
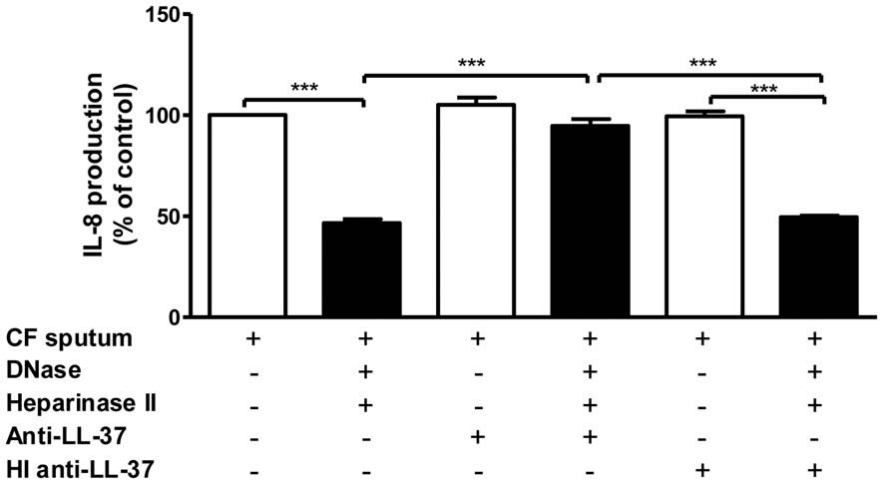
Increased LL-37 in DNase and heparinase II treated CF sputum inhibits IL-8 production from THP-1 monocytes. CF sputum samples (n = 12) were treated with Pulmozyme® DNase and/or heparinase II for 1 h at 37°C in the absence or presence of an LL-37 antibody or heat-inactivated (HI) LL-37 antibody as a negative control. Samples were incubated in LPS-immobilised 96-well microtiter plates (100 ng LPS/well) for 1 h at RT. Unbound peptides were removed by washing and THP-1 monocytes (1×10^6^/ml) were added to each well and incubated for 6 h at 37°C. The ability of released LL-37 to inhibit LPS-induced IL-8 production by THP-1 monocytes was determined by ELISA. Results are expressed as percentage of untreated control (100% = untreated CF sputum). *** *p*<0.001.

The increased level and concomitant LPS-binding activity of LL-37 caused by DNase and heparinase II treatment of CF sputa resulted in functional LPS-neutralising activity as shown in Figure 5. Our findings demonstrate that treatment of CF sputum with DNase and heparinase II was associated with a significant decrease in the amount of LPS-induced IL-8 produced by THP-1 cells when compared to untreated sputum. The inclusion of a specific anti-LL-37 antibody demonstrates that a significant proportion of this inhibition was mediated by liberated LL-37 present in the treated sputum, an effect that was negated when the antibody was heat-inactivated.

## Discussion

*P. aeruginosa* can take advantage of the compromised health of susceptible individuals including CF patients, and initiate an overwhelming host inflammatory response that also contributes significantly to morbidity and mortality of infected patients. *P. aeruginosa* LPS is a prominent factor in mediating both bacterial virulence and host responses [33]. In this study, we show that exogenous LL-37 inhibits *P. aeruginosa* LPS-induced IL-8 production. This potent knockdown of IL-8 production was associated with downregulation of the activity of a number of transcription factors such as NF-κB and also AP-1, STAT-1 and IRF-3 (data not shown). However, removal of exogenous LL-37 prior to LPS stimulation abolished these inhibitory effects. These findings suggest that a primary mechanism by which LL-37 inhibits LPS signalling is via neutralisation of LPS extracellularly. The ability of LL-37 to neutralise/detoxify LPS from a number of species, such as *E. coli*, *Burkholderia cepacia* and *Salmonella typhimurium*, has been well characterised [28]–[32]. Binding studies with LL-37 revealed that LL-37 binds with high affinity to *E. coli* LPS and with a stoichiometry of ∼1∶1 [13], [29]. Furthermore, LL-37 binds to LPS in solution and also when LPS is already bound to macrophages [29]. Published work to date suggests at least three mechanisms by which LL-37 may detoxify LPS: (1) LL-37 binds directly to LPS making it unavailable for LPS binding protein (LBP) and transfer to the primary LPS receptor – CD14; (2) LL-37 binds directly to CD14, and (3) LL-37 dissociates LPS oligomers preventing LPS from binding to LBP or CD14 [28]–[30], [51].

LPS possesses three structural domains, lipid A, core oligosaccharide, and O polysaccharides. The activation mechanism of LPS is initiated when lipid A binds to LPS-binding protein (LBP), which accelerates the binding of LPS to CD14. *P. aeruginosa* synthesizes more highly acylated lipid A structures during adaptation to the CF airway environment and the acylation state of LPS may alter TLR4-mediated responses [44]–[47]. In agreement with previous findings [44], THP-1 monocytes stimulated with LPS extracted from *P. aeruginosa* strains isolated from chronically-infected lungs of CF patients secreted higher amounts of IL-8 than cells stimulated with commercially available *P. aeruginosa* LPS at comparable concentrations demonstrating heightened activation of pro-inflammatory responses which is associated with more highly acylated forms of *P. aeruginosa* LPS. In addition to increased inflammation, these modifications are associated with resistance to cationic AMPs such as C18G and polymyxin [44]. However, in this study we show that LL-37 retains its ability to neutralise such highly acylated forms of LPS suggesting that this AMP could play a significant role in dampening LPS-induced inflammation in the CF lung. The ability of LL-37 to exert its antibacterial activity against such strains remains to be determined.

As an AMP, LL-37 displays antibacterial activity against *P. aeruginosa* with MICs of 1.3–16 µg/ml reported for various strains [11], [13]. Furthermore, a low concentration of LL-37 (0.5 µg/ml) potently inhibited the formation of *P. aeruginosa* biofilm [17]. These findings are of particular relevance to CF as *P. aeruginosa* is the most prevalent and destructive pulmonary pathogen in patients and biofilm formation is a crucial factor in chronic infection due to their inherent resistance to antimicrobial agents [17]. However, in the complex milieu of the CF lung, it appears that the antibacterial activity of LL-37 is diminished through interactions with various components of the lung environment such as DNA and GAGs [19], [26]. The number of neutrophils in the CF lung is estimated at least two orders of magnitude higher than patients with chronic bronchitis during a period of exacerbation [52]. In addition to LL-37, levels of GAGs, DNA and LPS in the CF lung also correlate with neutrophilia and/or neutrophil elastase activity [9], [10], [26], [52]–[54]. Furthermore, levels of GAGs such as heparin sulphate and chondroitin sulphate are markedly increased in CF bronchial tissue and sputum [48], [49], [55].

Polyanionic molecules such as GAGs and DNA are able to bind positively charged peptides including LL-37 in a charge-based interaction. Previous work has found that treatment of CF lung secretions with DNase and GAG lyases increased the levels of free LL-37, in addition to the bactericidal activity of samples [19], [26]. In concurrence with previous work, we show that the interaction of LL-37 with these molecules in the pulmonary environment of the CF lung also negates the ability of LL-37 to bind and thereby neutralise LPS. Our findings demonstrate decreased levels of LL-37 and LL-37-related LPS binding in CF sputum as a result of binding to GAGs and DNA. However, upon removal of GAGs and DNA by treatment with the lyase heparinase II and DNase, respectively, we were able to simultaneously and synergistically increase LL-37 levels and LPS-binding activity in CF sputum. In addition, this increase in LL-37 and LPS binding was associated with a significant decrease in LPS-induced IL-8 production when compared to untreated sputum. DNase (Pulmozyme®) is the most widely used mucolytic therapy in patients with CF, reducing the viscosity of lung secretions and aiding airway clearance. Degradation of DNA fragments also disrupts heterogeneous DNA-protein complexes, thereby liberating trapped cationic molecules like LL-37 [19].

The synergistic increase in both LL-37 levels and concomitant LL-37:LPS binding when sputum samples were treated with both DNase and heparinase II indicates that the use of DNase alone may liberate the peptide leaving it free to bind to GAGs, or vice versa. In contrast, co-treatment of heparinase II may serve to cooperatively enhance the activity of DNase, resulting in decreased total levels of anionic electrolytes and liberation of LL-37, which then remains free to exert its anti-endotoxin effects. These results suggest that a combination therapy of DNase and heparinase II could be explored as a possible treatment strategy for CF patients. The benefits of such a treatment could include a much greater decrease in lung secretion viscosity and thereby enhanced clearance, increased bacterial killing capacity within the mucosal layer, and a reduction in the inflammatory response generated by the massive LPS load in the CF lung. As GAGs are constituents of the lung extracellular matrix and therefore play an important role in lung function and homeostasis, alterations in their synthesis and degradation may affect lung pathology and understanding the changes in GAG expression that occur in lung diseases such as CF may lead to novel targets for pharmacological intervention [56]. Although GAG lyases from *F. heparinum* are presently being developed for therapeutic applications [57], their role in CF has not yet been explored.
